# Intimate partner violence in the Americas: a systematic review and reanalysis of national prevalence estimates

**DOI:** 10.26633/RPSP.2019.26

**Published:** 2019-03-20

**Authors:** Sarah Bott, Alessandra Guedes, Ana P. Ruiz-Celis, Jennifer Adams Mendoza

**Affiliations:** 1 Independent consultant to the Pan American Health Organization Independent consultant to the Pan American Health Organization WashingtonD.C. United States of America Independent consultant to the Pan American Health Organization, Washington, D.C., United States of America.; 2 Pan American Health Organization Pan American Health Organization WashingtonD.C. United States of America Pan American Health Organization, Washington, D.C., United States of America.

**Keywords:** Intimate partner violence, domestic violence, violence against women, surveys and questionnaires, Latin America, Caribbean region, Americas, Violencia de pareja, violencia doméstica, violencia contra la mujer, encuestas y cuestionarios, América Latina, Región del Caribe, Américas, Violência por parceiro íntimo, violência doméstica, violência contra a mulher, inquéritos e questionários, América Latina, Região do Caribe, Américas

## Abstract

**Objectives.:**

To describe what is known about the national prevalence of intimate partner violence (IPV) against women in the Americas across countries and over time, including the geographic coverage, quality, and comparability of national data.

**Methods.:**

This was a systematic review and reanalysis of national, population-based IPV estimates from 1998 – 2017 in the Americas. Estimates were reanalyzed for comparability or extracted from reports, including IPV prevalence by type (physical; sexual; physical and/or sexual), timeframe (ever; past year), and perpetrator (any partner in life; current/most recent partner). In countries with 3+ rounds of data, Cochran-Armitage and Pearson chi-square tests were used to assess whether changes over time were significant (*P <* 0.05).

**Results.:**

Eligible surveys were found in 24 countries. Women reported ever having experienced physical and/or sexual IPV at rates that ranged from 14% – 17% of women in Brazil, Panama, and Uruguay to over one-half (58.5%) in Bolivia. Past-year prevalence of physical and/or sexual IPV ranged from 1.1% in Canada to 27.1% in Bolivia. Preliminary evidence suggests a possible decline in reported prevalence of certain types of IPV in eight countries; however, some changes were small, some indicators did not change significantly, and a significant increase was found in the reported prevalence of past-year physical IPV in the Dominican Republic.

**Conclusions.:**

IPV against women remains a public health and human rights problem across the Americas; however, the evidence base has gaps, suggesting a need for more comparable, high quality evidence for mobilizing and monitoring violence prevention and response.

## RESUMEN

**Objetivos.:**

Describir lo que se sabe acerca de la prevalencia nacional de la violencia por parte de la pareja íntima (VPI) contra las mujeres en las Américas, en los diversos países y en el transcurso del tiempo, incluida la cobertura geográfica, calidad y comparabilidad de los datos nacionales.

**Métodos.:**

Se realizó una revisión sistemática y reanálisis de las estimativas nacionales de la VPI basadas en la población de 1998 a 2017 en las Américas. Las cifras se reanalizaron para comparabilidad o se extrajeron de los informes, incluida la prevalencia por tipo (física; sexual; o física y/o sexual), marco temporal (alguna vez; durante el último año) y perpetrador (cualquiera pareja en la vida; pareja actual/más reciente). En los países con 3+ rondas de datos, se aplicaron las pruebas de Cochran-Armitage y de ji cuadrada de Pearson para evaluar si los cambios en el transcurso del tiempo fueron significativos (*P <* 0,05).

**Resultados.:**

Se encontraron encuestas elegibles en 24 países. Las mujeres reportaron haber sufrido alguna vez violencia física y/o sexual por parte de la pareja íntima con tasas que variaron desde el 14% al 17% en Brasil, Panamá y Uruguay hasta más de la mitad (58,5%) en Bolivia. La prevalencia de violencia física y/o sexual por parte de la pareja íntima durante el último año varió desde 1,1% en el Canadá hasta 27,1% en Bolivia. La evidencia preliminar sugiere una posible disminución en la prevalencia reportada para ciertos tipos de VPI en ocho países; sin embargo, algunos cambios fueron pequeños, ciertos indicadores no se modificaron significativamente y se observaron incrementos significativos en la prevalencia reportada de violencia física por parte de la pareja íntima durante el último año en la República Dominicana.

**Conclusiones.:**

La VPI contra las mujeres sigue siendo un problema de salud pública y de derechos humanos en las Américas; sin embargo, la base de evidencia al respecto tiene deficiencias, lo que apunta a la necesidad de datos de mejor calidad y más comparables, a fin de movilizar y monitorear a la prevención y la respuesta ante la violencia.

## RESUMO

**Objetivos.:**

Descrever o que se sabe sobre a prevalência nacional da violência por parceiro íntimo (VPI) contra a mulher na Região das Américas, nos diferentes países e ao longo do tempo, incluindo cobertura geográfica, qualidade e comparabilidade de dados nacionais.

**Métodos.:**

Foi realizada uma revisão sistemática e reanálise das estimativas nacionais populacionais de VPI na Região das Américas no período de 1998 a 2017. As estimativas foram reanalisadas para fins de comparação ou obtidas de relatórios, incluindo a prevalência de VPI por tipo de violência (física; sexual; ou física e/ou sexual), ocorrência (alguma vez ou último ano) e agressor (qualquer parceiro na vida; parceiro atual ou mais recente). Nos países com mais de três ciclos de dados, os testes de Cochran-Armitage e qui-quadrado de Pearson foram usados para avaliar se as mudanças observadas ao longo do tempo foram significativas (*P <* 0,05).

**Resultados.:**

Pesquisas que cumpriam os requisitos do estudo foram identificadas em 24 países. O percentual de mulheres que informaram alguma vez terem sofrido VPI física e/ou sexual variou de 14% a 17% no Brasil, Panamá e Uruguai a mais da metade (58,5%) na Bolívia. A prevalência de VPI física e/ou sexual sofrida no último ano variou de 1,1% no Canadá a 27,1% na Bolívia. As evidências preliminares indicam uma possível redução na prevalência registrada de certos tipos de VPI em oito países. Porém, algumas mudanças foram pequenas, alguns indicadores não variaram significativamente e se observou um aumento significativo na prevalência informada de VPI física recente (último ano) na República Dominicana.

**Conclusões.:**

A VPI contra a mulher continua sendo um problema de saúde pública e uma questão de direitos humanos na Região das Américas. Porém, a base de evidências tem importantes lacunas, ressaltando a necessidade de dados de alta de qualidade e comparáveis para a mobilização e o monitoramento da prevenção e resposta à violência.

Violence against women (VAW) has been recognized as an important public health and human rights problem, both globally (1) and within the Region of the Americas (2). Intimate partner violence (IPV)—the most common form of VAW—has serious consequences for women’s health and wellbeing (3). In a 12-country analysis from the Region (4), large proportions of women who experienced IPV reported consequences such as physical injuries, chronic pain, anxiety, depression, and suicidal thoughts. In most countries, IPV was significantly correlated with lower age at first union, higher parity, and unintended pregnancy. IPV also has well-documented negative consequences for children and the broader society (5, 6).

In 2015, the United Nations (UN) Member States agreed to work toward eliminating VAW as part of 2030 Sustainable Development Goals (SDGs) (7). Member States of the Pan American Health Organization (PAHO) and the World Health Organization (WHO) made similar commitments as part of the PAHO 2015 Strategy and Plan of Action on VAW (8) and the WHO 2016 Global Plan of Action on Interpersonal Violence (9). Countries also agreed to strengthen data collection systems and measure SDG Indicator 5.2.1: the proportion of ever-partnered women and girls 15+ years of age subjected to physical, sexual, or psychological violence by a current or former intimate partner in the previous 12 months.

The number of countries with national IPV prevalence estimates has grown recently (10), but data are not always easy to find, comparable across countries or over time, or published in full (4). Databases of the UN Minimum Set of Gender Indicators (11) and the SDGs (12) have begun compiling national estimates, but these come primarily from Demographic and Health Surveys (DHS) and are often limited to IPV in the past 12 months, due to the formulation of SDG Indicator 5.2.1. Moreover, published estimates are often constructed in diverse ways regarding age, partnership status, and forms of violence (10). As a result, researchers and policy makers may lack access to comparable IPV estimates, even when data exist.

This study aims to describe what is known about the national prevalence of IPV against women in the Americas across countries and over time, including geographic coverage, quality, and comparability of data. A systematic review was carried out along with a comparative reanalysis of national, population-based, IPV prevalence estimates from PAHO Member States. In addition, changes over time were analyzed in countries with 3+ rounds of comparable data collection. To conclude, recommendations for improving measurement and dissemination are presented.

## MATERIALS AND METHODS

**FIGURE 1:**
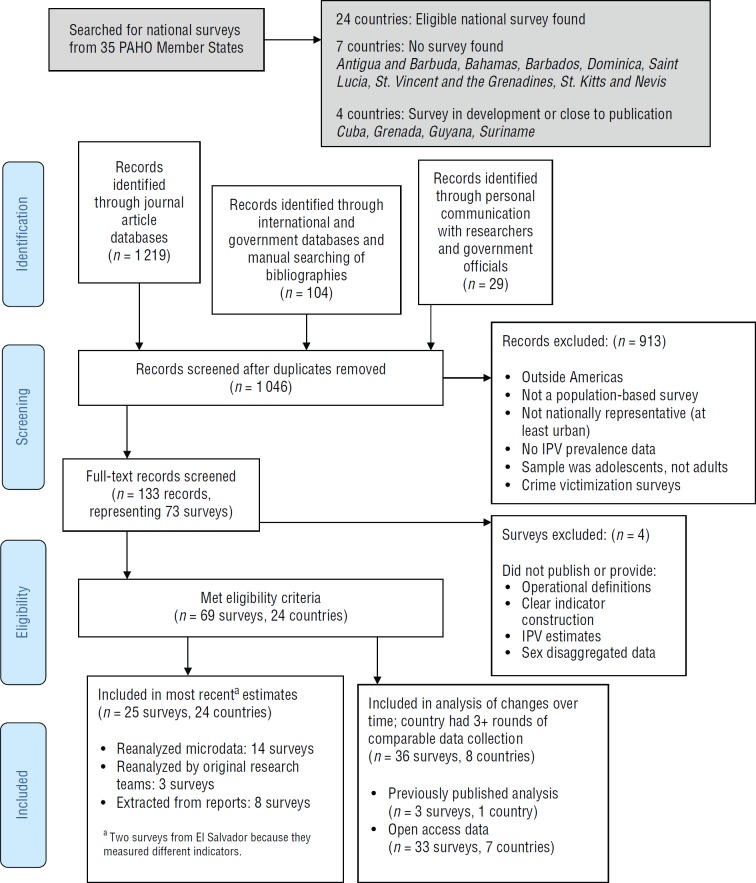
Flowchart of search and selection of surveys with national prevalence data on intimate partner violence (IPV) in the Americas

Per PRISMA guidelines (Figure 1), a systematic search for nationally representative, population-based surveys with IPV data from PAHO Member States was carried out in duplicate (by SB and AR) using terms such as ‘intimate partner violence,’ ‘violence against women,’ ‘domestic violence,’ ‘spouse abuse,’ ‘prevalence,’ ‘national survey,’ and country names. The search was performed on SciELO (Latin American and Caribbean Center on Health Sciences Information, São Paulo, Brazil), LILACS (Latin American and Caribbean Center on Health Sciences Information, PAHO/WHO, São Paulo, Brazil), PubMed Central (U.S. National Library of Medicine, Bethesda, Maryland, United States), and Google Scholar (Google Inc., Mountain View, California, United States); the databases of UN Women (13), SDGs (12), the Global Health Data Exchange (GHDx), Reproductive Health Surveys (RHS), DHS, and websites of national institutes of statistics (or similar agencies) in each country. Bibliographies of global and regional reviews were manually searched, and more than 100 researchers and government officials throughout the Region were contacted. After screening 1 046 records (once duplicates were removed), 133 records were selected for full text review. Eligibility was independently assessed by at least two authors (SB, AR, JM); differences of opinion were resolved by consensus among all authors.

*A priori* inclusion criteria were:

- Population-based, household or telephone surveys;
- Nationally representative (at least urban);
- From any PAHO Member State;
- Collected data on prevalence of IPV against women (not just adolescents);
- Published findings (at least online) in any language (English, French, Portuguese, or Spanish);
- Provided sufficient information on methods, operational definitions, and indicator construction to assess data quality (through personal communication if not published reports/questionnaires);
- Explicitly mentioned ‘partners’ in preambles or survey items measuring violence.

Eligible surveys collected data from January 1998 – December 2017 and published findings by 15 July 2018. The timeframe was expanded after work began, so database searches were updated in July 2018. Peer-reviewed journal publication was not required because national survey findings do not always reach journals in a timely manner, if at all. Urban-only studies were included to allow wider geographic coverage. Crime Victim Surveys (14) were excluded because they ask about violence by any perpetrator without explicitly mentioning partners—an approach known to underestimate prevalence (15). However, to ensure adequate geographic coverage, surveys that explicitly mentioned partners in preambles or survey questions were considered eligible, even if they asked about violence by ‘family members’ or ‘any man.’ If published reports provided inadequate information about methods or operational definitions, the information was sought directly from the authors/researchers. In four cases (16 – 19), the attempt to get more detail was not successful, so the surveys were excluded.

### Most recent IPV estimates

For the most recent eligible survey in each country, a secondary analysis of IPV prevalence was carried out by ***type*** (physical; sexual; or physical and/or sexual); ***timeframe*** (ever; or past year); and ***perpetrator*** (any partner in life; or current/most recent partner—‘current’ for women with a partner and ‘most recent’ for those separated, divorced, or widowed). Emotional/psychological IPV was not reanalyzed given the enormous diversity of measures across surveys in the Region and the lack of international consensus on definitions (3).

When datasets were open-access, estimates with confidence intervals (CIs) were reanalyzed for comparability (by JM or AR) using SPSS Statistics for Windows, Version 20 (IBM Corp., Armonk, New York, United States), SAS 9.1 (SAS Institute Inc., SAS Campus Drive, Cary, North Carolina, United States), or Stata Statistical Software^®^/MP14 (StataCorp LP, College Station, Texas, United States). Sample weights were applied to adjust for sampling design and non-response differentials when available. Analyses were reviewed by all authors and shared with original research teams, who often provided technical assistance. When microdata were unavailable or not feasible to reanalyze, the original researchers were contacted to request estimates reanalyzed for comparability. Otherwise, estimates were extracted from published reports in duplicate by at least two authors (SB, AR, JM) and confirmed with country teams when possible. CIs for estimates extracted from reports were calculated using Epitools epidemiological calculators (20), unless they were already published.

For comparability (within limits of datasets), reanalyzed indicators were constructed to align with most DHS surveys (4), including:

- Limiting the age range of women to 15 – 49 years;
- Classifying threats with a weapon as physical violence, not emotional;
- Retaining eligible women in denominators even if responses were missing for one or more violence questions;
- Limiting denominators to women who had ever married or cohabited with a partner (excluding women whose only partners in life were non-cohabiting);
- Producing separate estimates for violence by the current/most recent partner and for violence by any partner in life.

Other than threats with a weapon, operational definitions of physical violence were fairly consistent across surveys, and additional standardization seemed unnecessary. Sexual violence measures were more diverse, and without an international consensus on which acts to include (3), standardization did not seem advisable.

### Assessment of quality/risk of bias

Risk of bias was assessed using a checklist adapted from existing tools (21 – 23), informed by good practice guidelines for violence research (15, 24). Scoring was based on published reports, questionnaires, microdata, and personal communications with original researchers. Surveys received one point for any of the following unmet quality criteria:

- Population-based design;
- Nationally-representative sample (urban and rural; household survey);
- Justified sample size;
- Response rate > 66%;
- Weighted analysis;
- Valid/reliable IPV measures (partner and behaviorally specific measures);
- Estimates for both ever and past year;
- Estimates for both any partner in life and current/most recent partner;
- Dedicated violence survey (not a module);
- Clear adherence to WHO ethical guidelines (25) regarding privacy, consent, and confidentiality (only one woman per household);
- Denominators composed of ever-partnered women (however defined) of reproductive age.

‘Most recent’ estimates also received a point if they were ≥ 8 years old (i.e., implemented in or before 2010). Scoring was performed in duplicate (SB, AR), with discrepancies resolved by consensus among authors.

### Changes over time

To explore changes over time, the search identified countries with 3+ rounds of comparable data collection (1998 – 2017). Estimates were reanalyzed using open access microdata or extracted from Kishor and Johnson (26), known to have used comparable indicator construction.

To obtain three comparable data points, past year estimates from Guatemala and Mexico were limited to women married or cohabiting at the time of the interview. Additionally, sexual IPV estimates from Guatemala and Nicaragua were limited to forced sex, excluding ‘unwanted sex due to fear of what her partner might do if she refused’ (which was not measured in all survey years). Indicator construction of all other estimates used to analyze changes over time matched construction used to analyze the ‘most recent’ prevalence estimates.

Physical IPV and sexual IPV, ever and past 12 months, were analyzed separately in case they changed at different rates or in different directions. Using XLSTAT 2017 (Addinsoft, Paris, France), the significance (*P <* 0.05) of changes over time was assessed with the Cochran-Armitage trend test, which has been used widely for this purpose (27). Pearson chi-square was used to test significance of differences between first and last data points. Surveys (from Mexico) that weighted estimates expanded to population size were tested with both weighted and unweighted data (producing the same results).

## RESULTS

The search identified 69 eligible surveys from 24 countries in the Americas. Four additional countries (Cuba, Grenada, Guyana, and Suriname) had potentially eligible surveys in development or in press as of July 2018.

Table 1 presents study characteristics and sources for the 25 ‘most recent’ eligible surveys from each country (29 – 48), including two from El Salvador that measured different indicators. As noted in the table, 15 surveys were dedicated to violence; 10 used a violence module embedded in a larger survey. Estimates from 14 surveys were reanalyzed using open access microdata; reanalyzed estimates from three surveys were obtained directly from original researchers; and published estimates from eight surveys were extracted from reports. Most (21 of 25) were household surveys, except for telephone surveys in Argentina, Brazil, Canada, and the United States. Many used instruments from international research programs, such as DHS, RHS, the International Violence Against Women Survey, or the WHO Multi-Country Study. Five used instruments modeled on the *Encuesta Nacional sobre la Dinámica de las Relaciones en los Hogares* from Mexico (28). National statistics offices carried out some surveys; civil society researchers implemented others; but most involved collaboration between government and civil society (not shown). Most estimates were limited to women 15 – 49 years of age who ever married/cohabited, but fully standardized denominators were not always possible, especially for estimates extracted from reports.

The quality assessment of ‘most recent’ estimates identified risks of bias such as: incomplete national representativeness (two urban-only surveys; four that excluded women unreachable by phone; two with other limits to national coverage); inadequate sample size justification (three surveys); response rates unreported or ≤ 66% (eight surveys); unweighted estimates (four surveys); IPV questions not partner or behaviorally-specific (four surveys); nonstandard denominators (eight surveys); and estimates ≥8 years old (four surveys). Many surveys did not clearly adhere to WHO ethical guidelines. Fourteen did not clearly remind women that they were free to refuse participation, stop the interview, or decline to answer violence questions; and one survey (Colombia 2015) interviewed all adults—not just one woman—in the home about violence.

Table 2 presents the secondary analysis of ‘most recent’ national IPV prevalence estimates, by partner, type of violence, and timeframe, along with risk of bias score and rating. Sixteen surveys were classified as low risk of bias; six as moderate; and three as high. The proportion of women who reported physical and/or sexual IPV* ever* ranged from about 14% – 17% in Brazil, Panama, and Uruguay to more than half (58.5%) in Bolivia. In 14 (a majority of) countries, prevalence ranged from one-fourth to one-third; and in five countries (Bolivia, Colombia, Costa Rica, Ecuador, and the United States), prevalence exceeded one-third. Reported prevalence of physical and/or sexual IPV *in the past year* ranged from 1.1% in Canada to 27.1% in Bolivia.

In eight countries that measured violence (*ever*) both by *any partner in life* and by the *current/most recent partner*, the former was significantly higher than the latter for both physical and sexual IPV. In Uruguay 2013, estimated prevalence of physical and/or sexual violence by any partner was twice as high as violence by the current/most recent partner (16.8% vs. 7.6%). In contrast, *past yea*r estimates of violence by the current/most recent partner were similar, if not identical, to any partner in all countries with data, and differences never exceeded CIs.

### Changes in reported prevalence levels over time

Eight countries (Canada, Colombia, the Dominican Republic, Guatemala, Haiti, Mexico, Nicaragua, and Peru) had 3+ rounds of eligible data collection over 15 – 20 years using a similar instrument. Canadian datasets were not open access, but a previously published analysis (33) documented a significant (*P <* 0.05) decline in physical and/or sexual IPV prevalence, both for the 5 years preceding the survey: 7.2% (2004), 6.4% (2009), 3.5% (2014); and for the previous year: 2.2% (2004), 1.9% (2009), 1.1% (2014).

**TABLE 1:** Sources and methodological characteristics of most recent eligible national IPV estimates from the Americas, by survey

**Country, year**	**Source **	**Instrument**	**Method**	**Women’s age and partnership** (if not 15-49, ever married/cohabited)	**Possible risk of bias^a^**
Argentina 2015	Report (29)	IVAWS	Dedicated, telephone	18-69; All women	2a,4,5,8,11
Belize 2015	Report (30)	WHO	Dedicated, household	18-64; Ever had romantic partner^b^	2c,3,5,8,11
Bolivia 2016	Reanalysis^c^ (31)	Based on ENDIREH	Dedicated, household		4,8,10
Brazil 2017	Research Team (32)	–	Dedicated, telephone	16+; All women	2a,3,4,6ab,8,10,11
Canada 2014	Report (33)	–	Dedicated, mixed telephone/household	15+; Past 5 years married, cohabited or in contact with an ex; included male and female partners	2a,4,7,8,10,11
Chile 2016/17	Report (34)	–	Dedicated, household	15-65; All women/currently had romantic partner^d^	2b,4,6a,7,8,10,11
Colombia 2015	Reanalysis (35)	DHS	Module, household		8,9,10
Costa Rica 2003	Research Team (36)	IVAWS	Dedicated, household	18-69; Ever had romantic partner^b^	4,6a,8,11,12
Dominican Republic 2013	Reanalysis (35)	DHS	Module, household		9,10
Ecuador 2011	Reanalysis^c^ (37)	Based on ENDIREH	Dedicated, household		10
El Salvador 2017	Reanalysis^c^ (38)	Based on ENDIREH	Dedicated, household	Ever had romantic partner^b^	8,10
El Salvador 2013/14	Reanalysis^c^ (39)	WHO	Dedicated, household		2c,5
Guatemala 2014/15	Reanalysis (35)	DHS	Module, household		9
Haiti 2016/17	Reanalysis (35)	DHS	Module, household		9
Honduras 2011	Reanalysis (35)	DHS	Module, household		9,10
Jamaica 2016	Research team (40)	WHO	Dedicated, household	Ever married, cohabited, had ‘regular’ (visiting) partner^b^	8
Mexico 2016	Reanalysis^c^ (28)	ENDIREH	Dedicated, household		10
Nicaragua 2011/12	Reanalysis (41)	DHS	Module, household		8,9
Panama 2009	Report (42)	DHS	Module, household		8,9,10,12
Paraguay 2008	Reanalysis (43)	RHS	Module, household	15-44	8,9,12
Peru 2017	Reanalysis (44)	DHS	Module, household		8,9,10
Trinidad & Tobago 2017	Report (45)	WHO	Dedicated, household	15-64; Ever had romantic partner^b^	5,8,11
Uruguay 2013	Reanalysis^c^ (46)	Based on ENDIREH	Dedicated, household	Included male and female partners	2b,4,10
USA 2010/12	Report (47)	–	Dedicated, telephone	18+; All women.	2a,6a,8,11
Venezuela 2010	Report (48)	DHS	Module, household		2c,3,4,8,9,10,12

a ***Risk of bias:*** 2a. Excluded women unreachable by phone; 2b. Urban only; 2c. Other barrier to national coverage; 3. Inadequate/unclear sample size justification; 4. Response rate unreported or ≤66%; 5. Estimates unweighted; 6a. IPV questions not partner specific; 6b. IPV questions not behaviorally specific; 7. Did not measure both ever and past year; 8. Did not produce estimates for both any partner and current/most recent; 9. Module, not dedicated survey; 10. Did not clearly adhere to WHO ethical guidelines; 11. Non-standardized denominator, not reproductive age and/or ever-partnered (however defined); 12. Estimates ≥ 8 years old.

b Included women who ever had a non-cohabiting partner such as a boyfriend.

c Reanalyzed by authors with assistance from original research team.

d Physical IPV: all women; sexual IPV: current had romantic partner (not necessarily cohabiting).

IVAWS: International Violence Against Women Survey; WHO: World Health Organization; ENDIREH: Encuesta Nacional sobre la Dinámica de las Relaciones en los Hogares; DHS: Demographic and Health Survey; RHS: Reproductive Health Survey

**FIGURE 2:**
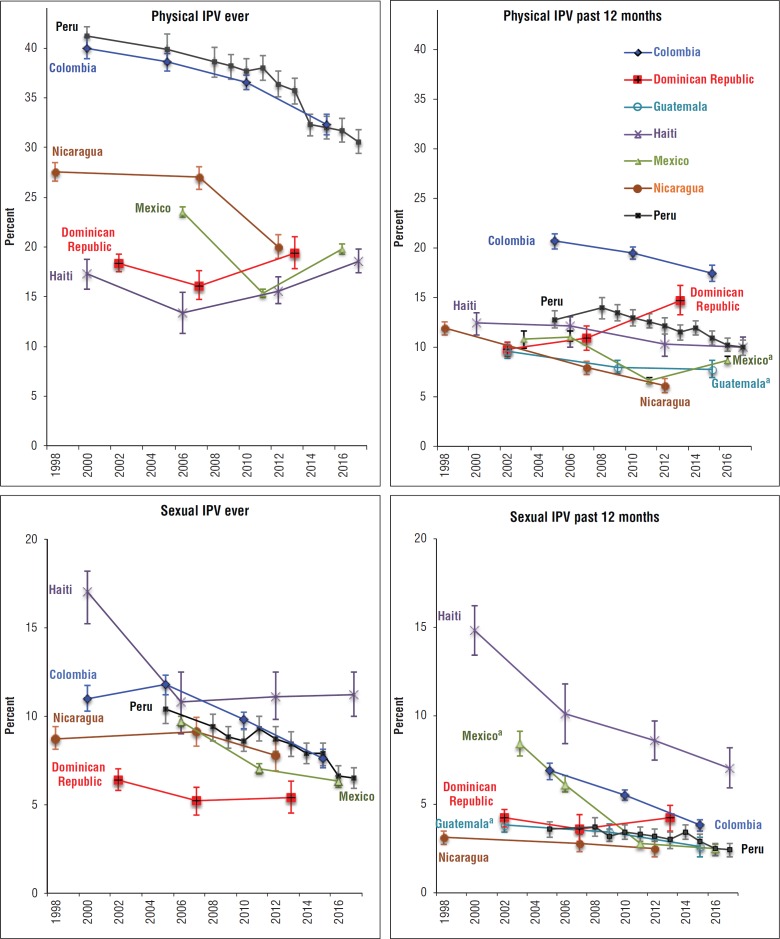
Percentage of ever-partnered^a^ women aged 15-49 who reported physical or sexual intimate partner violence (IPV), ever and past 12 months, by country and year^b^

***Sources:*** Provided in Table 3 along with additional notes.

^a^ Past year estimates from Guatemala and Mexico were for currently partnered women; all other estimates were for ever partnered women.

^b^ Surveys that straddled more than one year are plotted as the later year.

Seven countries had 3+ rounds of comparable, open access data, tested for significance with Cochran-Armitage, unless noted (Table 3 and Figure 2). Reported prevalence of *past year physical IPV* increased significantly in the Dominican Republic (*P <* 0.0001). All other countries documented a significant downward change over time, including Colombia (*P <* 0.0001), Guatemala (*P <* 0.001), Haiti (*P <* 0.001), Mexico (*P <* 0.0001), and Peru (*P <* 0.0001). In Nicaragua, past year physical IPV declined by nearly one-half (11.9% to 6.1%, *P <* 0.0001). In Mexico and Peru, declines were not consistent across all data points (prevalence rose, then fell).

Reported prevalence of *past year sexual IPV* declined significantly in Colombia (*P <* 0.0001), Guatemala (*P <* 0.001), Haiti (*P <* 0.0001), Mexico (*P <* 0.0001), Nicaragua (*P <* 0.05), and Peru (*P <* 0.0001), but was unchanged in the Dominican Republic. In Mexico, past year sexual IPV declined by more than two-thirds (from 8.0% to 2.5%) from 2003 – 2016.

In some cases, past year physical and sexual IPV prevalence changed in different directions or at different rates. In the Dominican Republic, past year physical IPV increased by almost 50% (9.8% to 14.7%) from 2002 – 2013, while sexual IPV remained unchanged. In Haiti, past year sexual IPV estimates declined by more than one-half (from 14.8% to 7.0%), while physical IPV declined by one-fifth (12.5% to 10.0%).

Reported prevalence of *physical IPV ever* declined significantly (*P <* 0.0001) over time in four countries: falling by one-fifth (40.0% to 32.3%) in Colombia; one-fourth (41.2%, to 30.6%) in Peru; nearly one-third (27.6% to 20.0%) in Nicaragua; and one-seventh (23.5% to 19.8%) in Mexico. In the Dominican Republic, changes were neither unidirectional nor significant. In Haiti, Cochran-Armitage suggested a significant (*P <* 0.05) upward trajectory over time, but the increase from 2000 – 2017 was not significant per Pearson’s chi-square.

The reported prevalence of *sexual IPV ever* declined significantly over time in all countries with data. In Colombia, the Dominican Republic, Haiti, Nicaragua, and Peru, prevalence did not decline consistently across all data points (sometimes rising before falling), but the overall downward trajectory was significant per Cochran-Armitage (*P <* 0.0001 except for the Dominican Republic [*P <* 0.05] and Nicaragua [*P <* 0.001]).

**TABLE 2:** Percentage of women who reported physical and/or sexual IPV, ever and past 12 months (among women aged 15-49 who had ever married or cohabited unless noted in Table 1)

**Country, year**	**Partner**	**Physical and/or sexual**				**Sexual**				**Physical**				**N**	**Risk of bias**	
		**Ever**		**Past year**		**Ever**		**Past year**		**Ever**		**Past year**				
		%	CI	%	CI	%	CI	%	CI	%	CI	%	CI	Unweighted	No.	Rating
Argentina 2015	Any	26.9	24.4-29.4	2.7	1.8-3.6	3.9	2.8-5.0	0.2	0.0-0.4	26.5	24.1-29.0	2.7	1.8-3.6	1 221	5	Mod
Belize 2015	Any	22.2	18.5-25.8	—	—	6.9	4.8-9.2	—	—	21.9	18.3-25.6	—	—	501	5	Mod
Bolivia 2016	CMR	58.5	56.8-60.3	27.1	25.5-28.8	34.6	32.9-36.4	16.3	14.9-17.7	52.4	50.6-54.2	21.4	20.2-23.3	4 149	3	Low
Brazil 2017	Any	16.7	14.2-19.6	3.1	2.1-4.6	2.4	1.5-3.8	0.7	0.3-1.8	16.1	13.6-19.0	2.7	1.8-4.1	1 116	7	High
Canada 2014	Any	—	—	1.1	—	—	—	—	—	—	—	—	—	a	6	Mod
Chile 2016/17	Any	—	—	—	—	6.7	—	2.1	—	—	—	2.7	2.3-3.1	6 824	7	High
Colombia 2015	CMR	33.3	32.2-34.3	18.3	17.5-19.2	7.6	7.1-8.1	3.8	3.5-4.1	32.3	31.3-33.4	17.5	16.6-18.3	24 862	3	Low
Costa Rica 2003	Any	35.9	32.6-39.2	7.8	6.0-9.6	15.3	12.8-17.8	2.5	1.5-3.6	33.4	30.2-36.6	6.9	5.1-8.6	822	5	Mod
Dominican Republic 2013	Any	28.5	26.7-30.2	16.0	14.5-17.5	9.3	8.2-10.4	4.4	3.7-5.2	27.3	25.6-29.1	15.1	13.6-16.6	5 803	2	Low
	CMR	20.4	18.8-22.0	15.6	14.2-17.1	5.4	4.5-6.3	4.2	3.5-4.9	19.4	17.8-21.0	14.7	13.3-16.2			
Ecuador 2011	Any	40.4	38.7-42.1	—	—	14.3	13.1-15.4	4.0	3.4-4.8	38.6	37.0-40.4	—	—	9 131	1	Low
	CMR	35.5	33.8-37.2	10.8	9.7-11.9	10.2	9.2-11.2	3.9	3.2-4.5	34.4	32.7-36.0	9.3	8.2-10.3			
El Salvador 2017	CMR	14.3	11.8-16.9	5.9	4.1-7.7	5.0	3.4-6.7	2.0	0.9-3.1	13.7	11.1-16.2	5.4	3.7-7.2	2 127	2	Low
El Salvador 2013/14	Any	24.7	20.6-28.8	6.7	4.9-8.6	11.9	8.7-15.2	3.2	1.9-4.6	20.6	17.1-24.6	4.9	3.4-6.4	741	2	Low
	CMR	15.7	11.8-19.6	—	—	7.7	4.9-10.7	—	—	12.0	9.1-15.6	—	—			
Guatemala 2014/15	Any	21.2	19.9-22.6	8.5	7.6-9.5	7.1	6.2-7.9	2.6	2.0-3.1	20.4	19.1-21.8	7.9	7.1-8.8	6 512	1	Low
	CMR	18.0	16.7-19.3	8.5	7.6-9.5	5.2	4.5-5.9	2.6	2.0-3.1	17.3	16.1-18.6	7.9	7.0-8.7			
Haiti 2016/17	Any	26.0	24.2-27.8	13.9	12.5-15.4	14.0	12.5-15.5	7.2	6.1-8.4	21.3	19.6-23.0	10.1	8.9-11.3	4 322	1	Low
	CMR	23.5	21.7-25.3	13.8	12.3-15.2	11.2	9.8-12.5	7.0	5.9-8.2	18.6	17.0-20.2	10.0	8.7-11.2			
Honduras 2011/12	Any	27.8	26.7-28.9	11.0	10.3-11.7	10.9	10.1-11.6	3.3	2.8-3.7	25.9	24.8-27.0	10.0	9.3-10.7	12 494	2	Low
	CMR	21.6	20.6-22.6	10.9	10.2-11.6	6.5	5.9-7.1	3.2	2.8-3.6	20.2	19.2-21.2	10.0	9.3-10.7			
Jamaica 2016	Any	28.1	24.8-31.3	8.6	6.5-10.6	7.6	5.7-9.5	2.5	1.4-3.6	25.6	22.4-28.7	7.1	5.2-8.9	723	1	Low
Mexico 2016	Any	24.6	24.0-25.1	—	—	7.8	7.4-8.1	—	—	23.3	22.8-23.9	—	—	60 040	1	Low
	CMR	21.0	20.5-21.5	9.5	9.1-9.9	6.3	6.0-6.6	2.7	2.5-2.9	19.8	19.3-20.3	8.6	8.3-9.0			
Nicaragua 2011/12	Any	22.5	21.3-23.8	7.5	6.7-8.3	10.1	9.2-10.9	3.5	3.0-4.0	20.0	18.9-21.2	6.1	5.4-6.8	12 065	2	Low
Panama 2009	CMR	14.4	13.5-15.3	10.1	9.3-10.9	3.2	2.8-3.7	2.7	2.3-3.1	13.8	12.9-14.7	9.2	8.5-10.0	5 831	4	Mod
Paraguay 2008	Any	20.4	18.8-22.0	8.0	6.9-9.0	8.9	7.8-10.0	3.3	2.7-3.9	17.9	16.3-19.4	6.7	5.8-7.6	4 414	3	Low
Peru 2017	CMR	31.2	30.5-31.9	10.6	10.2-11.0	6.5	6.2-6.9	2.4	2.2-2.6	30.6	29.9-31.3	10.0	9.6-10.4	21 454	3	Low
Trinidad & Tobago 2017	Any	30.2	27.5-33.0	5.7	4.4-7.1	10.5	8.7-12.3	0.9	0.4-1.5	28.3	25.6-31.0	5.1	3.8-6.4	1 079	3	Low
Uruguay 2013	Any	16.8	14.6-19.0	3.1	2.1-4.1	6.6	5.3-8.0	0.6	0.2-1.1	15.7	13.6-17.7	2.9	2.0-3.9	1 560	3	Low
	CMR	7.6	6.1-9.0	2.8	1.8-3.7	2.4	1.6-3.2	0.6	0.2-1.0	7.0	5.6-8.4	2.6	1.8-3.5			
USA 2010/12	Any	37.3^b^	36.3-38.3	6.6^b^	6.0-7.1	16.4	15.6-17.1	2.1	1.8-2.4	32.4	31.5-33.4	3.9	3.5-4.4	22 590	4	Mod
Venezuela 2010	CMR	17.9	—	12.2	—	4.7	—	3.3	—	17.5	—	11.7	—	a	7	High

Any: any partner in life. CMR: Current or most recent partner. –: unavailable. Mod: Moderate.

a Unweighted denominator unavailable; full sample size: 17 966 (Canada); 3 793 (Venezuela).

b Also included stalking.

**TABLE 3:** Percentage of women who reported physical or sexual IPVa ever and past 12 months (among women aged 15-49 who ever married or cohabited unless noted) by country and year of survey

**Country**	**Year**	**Source**	**Physical**		**Sexual**		**N**	**Risk of bias**	
			**Ever**	**Past year**	**Ever**	**Past year**			
			%	%	%	%	Unweighted	No.	Rating
**Colombia**			Declined***	Declined***	Declined***	Declined***			
	2000	(27)	40.0	—	11.0	—	7 716	4	Mod
	2005	(35)	38.6	20.7	11.8	6.9	25 620	3	Low
	2010	(35)	36.6	19.5	9.8	5.5	34 624	3	Low
	2015	(35)	32.3	17.5	7.6	3.8	24 862	3	Low
**Dominican Republic**			**ns**	Increased***	Declined*	**ns**			
	2002	(27)	18.4	9.8	6.4	4.2	7 435	3	Low
	2007	(35)	16.1	10.9	5.2	3.6	8 438	3	Low
	2013	(35)	19.4	14.7	5.4	4.2	5 803	2	Low
**Guatemala**				Declined**		Declined**			
(currently married/cohabiting)	2002	(43)		9.6		3.8^c^	6 381	4	Mod
	2008/9	(43)	b	8.0	b	3.4^c^	11 416	1	Low
	2014/15	(35)		7.8		2.6^c^	6 512	1	Low
**Haiti**			Increased*^d^	Declined**	Declined***	Declined***			
	2000	(27)	17.3	12.5	17.0	14.8	2 592	2	Low
	2005/6	(35)	13.4	12.1	10.8	10.1	2 680	2	Low
	2012	(35)	15.6	10.3	11.1	8.6	6 650	1	Low
	2016/17	(35)	18.6	10.0	11.2	7.0	4 322	1	Low
**Mexico**				Declined***		Declined***			
(ever married/cohabiting)	2006	(28)	23.5		9.7		69 228	2	Low
	2011	(28)	15.4	b	7.0	b	75 405	2	Low
	2016	(28)	19.8		6.3		60 040	1	Low
**Mexico**				Declined***		Declined***			
(currently married/cohabiting)	2003	(28)		10.8		8.4	26 538	3	Mod
	2006	(28)	e	11.0	e	6.1	63 048	2	Low
	2011	(28)		6.6		2.8	63 273	2	Low
	2016	(28)		8.7		2.5	52 265	1	Low
**Nicaragua**			Declined***	Declined***	Declined**	Declined*			
	1998	(27)	27.6	11.9	8.7^c^	3.0^c^	8 508	3	Low
	2006/7	(43)	27.0	8.0	9.1^c^	2.8^c^	11 393	2	Low
	2011/12	(41)	20.0	6.1	7.8^c^	2.5^c^	12 065	2	Low
**Peru**			Declined***	Declined***	Declined***	Declined***			
	2000	(35)	41.2	—	—	—	18 196	5	Mod
	2004/6	(35)	39.9	12.8	10.4	3.6	10 233	3	Low
	2007/8	(35)	38.6	14.0	9.4	3.7	12 572	3	Low
	2009	(44)	38.2	13.5	8.8	3.2	13 781	3	Low
	2010	(44)	37.7	13.0	8.6	3.4	12 880	3	Low
	2011	(44)	38.0	12.6	9.3	3.3	12 898	3	Low
	2012	(44)	36.4	12.1	8.7	3.2	13 483	3	Low
	2013	(44)	35.7	11.5	8.4	3.0	13 174	3	Low
	2014	(44)	32.3	11.9	7.9	3.4	14 066	3	Low
	2015	(44)	32.0	10.9	7.9	2.9	22 696	3	Low
	2016	(44)	31.7	10.2	6.6	2.5	21 115	3	Low
	2017	(44)	30.6	10.0	6.5	2.4	21 454	3	Low

* =p<0.05; **=p<0.001; ***=p<0.0001; ns = not significant; per Cochran-Armitage; Mod: moderate.

a By current/most recent partner except in Nicaragua, where estimates were for any partner.

b Three comparable data points were unavailable.

c Limited to forced sex to produce three comparable data points.

d Statistically significant per Cochran-Armitage, but not Pearson’s chi square.

e Measured but not analyzed.

## DISCUSSION

This systematic review found that a majority (24 of 35) of PAHO Member States had national (at least urban), population-based IPV prevalence estimates that met inclusion criteria, with more forthcoming. Estimates from Costa Rica, Panama, Paraguay, and Venezuela were 8+ years old, but most countries had more recent estimates (past 5 years). Data quality varied, but the majority (15 of 24 countries) had national surveys with ≤ 3 risks of bias.

The analysis of ‘most recent’ estimates suggests that IPV against women remains widespread across the Americas. Reported prevalence of physical and/or sexual IPV ***ever*** varied from about 1 in 7 ever-partnered women in Brazil, Panama, and Uruguay to over 50% in Bolivia. Past year prevalence ranged from 1% in Canada to 27% in Bolivia. Generally, these align with WHO estimates (3) that nearly one-third (29.8%) of ever-partnered women in Latin America and the Caribbean have ever been physically and/or sexually abused by an intimate partner; however, this review highlights wide variations by country.

Many estimates in this analysis differ from those in published country reports due to differences in indicator construction (e.g., age range and partnership status of women included in denominators, treatment of missing values, or classification of threats with a weapon). Unfortunately, reports often failed to identify whether estimates were for any partner or for the current/most recent partner; forms and/or timeframes of violence; or characteristics of women in denominators. Sometimes, this information had to be obtained from the questionnaires or through personal communication with researchers. This analysis found preliminary evidence that certain types of IPV may have declined in Canada, Colombia, the Dominican Republic, Guatemala, Haiti, Mexico, Nicaragua, and Peru over the past 15 – 20 years. However, most countries had only three data points; some changes were small, some indicators did not change significantly, and a possible increase in physical IPV was found in the Dominican Republic (past year) and Haiti (ever). Changes in past year prevalence may reflect recent changes in levels of violence, while changes in lifetime prevalence may reflect longer term changes in the lifelong experiences of younger women compared with older cohorts of women aging out of samples.

**Limitations**. This analysis had numerous limitations. The focus on national estimates excluded high quality subnational surveys such as WHO surveys from Brazil and Peru that produced higher prevalence estimates than national surveys from the same time period (4). National estimates also obscure subnational variations (documented in virtually all national reports), and variations by women’s sociodemographic characteristics, such as age, education, employment, and wealth (4).

While this study provides a more comparative view across the Region than previously available, many limits to comparability remain. Underlying datasets were based on diverse measures of violence (questions and acts). Partnership status of women in denominators could not be standardized for all countries. Therefore, caution is required when comparing IPV estimates across countries, especially when comparing estimates of violence by the current/most recent partner with estimates of violence by any partner in life. Women often have more than one partner in life, and estimates restricted to violence by a single partner will—by definition—fail to capture abuse by partners prior to the current/most recent relationship.

Different age ranges of women in denominators pose another barrier to comparability across surveys. While SDG Indicator 5.2.1 uses a denominator of women 15+ years of age, metadata (10) acknowledge that most national estimates are for women of reproductive age (15 – 49 years). Important questions remain about how IPV prevalence among women of reproductive age compares with all women over 15 years of age.

Survey methods and risk of bias also varied. Telephone surveys excluded women unreachable by phone and may have achieved different disclosure rates than face-to-face interviews. The 25 ‘most recent’ surveys were conducted over a 15-year period (2003 – 2017). More than half of recent surveys did not clearly adhere to WHO ethical guidelines. Even when surveys used similar methods and operational definitions, differences in field procedures, interviewer selection, and training may have affected data quality (49). Methodological differences may explain why a national 2012 survey from Brazil (50) reported a past year prevalence of physical and/or sexual IPV of 6.3%, twice the 3.1% rate from the 2017 Brazil survey in Table 2. When scored for quality, the 2012 survey scored as ‘moderate’ risk while 2017 scored as ‘high.’ The 2012 survey used census-based, multistage cluster sampling and face-to-face interviews rather than telephone methods; measured violence with behavior-specific questions (which the 2017 survey did not); and limited denominators to currently married or cohabiting women (rather than all women).

Caution is also recommended when interpreting declines in reported prevalence over time given that prevalence sometimes rose before it fell and only four countries had more than three data points, the minimum number needed to draw preliminary inferences about change. Small changes in questionnaire design across surveys may also have affected estimates. For example, if Guatemalan and Nicaraguan sexual IPV estimates had included ‘unwanted sex due to fear’ (measured in one year but not others), estimates would have risen before falling due to a measurement artifact. Similarly, interviewer selection, skills, and training quality may have varied across surveys, possibly affecting disclosure and data quality (49). In some countries, survey methods improved over time, including better adherence to WHO ethical guidelines regarding privacy in Nicaragua and confidentiality in Peru. Finally, women’s willingness to disclose violence to interviewers may have changed over time due to changes in gender norms, social stigma attached to violence, and/or exposure to mass media messages about violence—whether or not actual prevalence changed.

## Conclusions

Population-based evidence confirms that IPV against women remains a widespread public health and human rights problem in the Americas. Reported prevalence rates declined significantly in several countries; however, some did not, some changes were small, and others rose over time, suggesting a need for more and sustained investment in violence prevention and response.

This review also suggests a need for greater geographic coverage, quality, and comparability of national IPV estimates. Ideally, surveys would measure violence both by any partner in life and by the current/most recent partner; use partner and behavior-specific surveys questions; construct indicators per UN guidelines (24); and disaggregate data for women 15 – 49 years of age, ever married/cohabited. Researchers need to label indicators clearly for type, timeframe, and perpetrators of violence, and improve adherence to WHO ethical guidelines, particularly informed consent. A stronger evidence base (that meets international ethical standards) could help countries raise awareness, mobilize evidence-informed programs and policies, and monitor progress toward SDGs.

## Author contributions.

AG took a lead role in the conception and design. SB and ARC carried out the search for eligible datasets. All authors contributed to data analysis plans and interpretation. JM and ARC conducted the statistical analysis. SB led the writing process. All authors reviewed and approved the final version.
